# Serum Albumin Domain Structures in Human Blood Serum by Mass Spectrometry and Computational Biology*[Fn FN2]

**DOI:** 10.1074/mcp.M115.048504

**Published:** 2015-09-18

**Authors:** Adam Belsom, Michael Schneider, Lutz Fischer, Oliver Brock, Juri Rappsilber

**Affiliations:** From the ‡Wellcome Trust Centre for Cell Biology, University of Edinburgh, Edinburgh EH9 3BF, United Kingdom;; §Robotics and Biology Laboratory, Technische Universität Berlin, 10587 Berlin, Germany;; ¶Department of Bioanalytics, Institute of Biotechnology, Technische Universität Berlin, 13355 Berlin, Germany.

## Abstract

Chemical cross-linking combined with mass spectrometry has proven useful for studying protein-protein interactions and protein structure, however the low density of cross-link data has so far precluded its use in determining structures *de novo*. Cross-linking density has been typically limited by the chemical selectivity of the standard cross-linking reagents that are commonly used for protein cross-linking. We have implemented the use of a heterobifunctional cross-linking reagent, sulfosuccinimidyl 4,4′-azipentanoate (sulfo-SDA), combining a traditional sulfo-*N*-hydroxysuccinimide (sulfo-NHS) ester and a UV photoactivatable diazirine group. This diazirine yields a highly reactive and promiscuous carbene species, the net result being a greatly increased number of cross-links compared with homobifunctional, NHS-based cross-linkers. We present a novel methodology that combines the use of this high density photo-cross-linking data with conformational space search to investigate the structure of human serum albumin domains, from purified samples, and in its native environment, human blood serum. Our approach is able to determine human serum albumin domain structures with good accuracy: root-mean-square deviation to crystal structure are 2.8/5.6/2.9 Å (purified samples) and 4.5/5.9/4.8Å (serum samples) for domains A/B/C for the first selected structure; 2.5/4.9/2.9 Å (purified samples) and 3.5/5.2/3.8 Å (serum samples) for the best out of top five selected structures. Our proof-of-concept study on human serum albumin demonstrates initial potential of our approach for determining the structures of more proteins in the complex biological contexts in which they function and which they may require for correct folding. Data are available via ProteomeXchange with identifier PXD001692.

High-resolution structures of proteins are essential for understanding cellular processes. Determining protein structures, however, is difficult: protein stability, purity, quantity, and solubility critically affect success. Nuclear magnetic resonance (NMR)^1^ spectroscopy can only be applied to proteins of limited size, whereas x-ray crystallography necessitates prior crystallization of the protein. These conditions make structure determination challenging for many proteins of biological relevance. This includes especially proteins that contain intrinsically unstructured or long coiled-coil regions, proteins associated to a membrane (1, 2) or parts of multi-protein complexes (3). New developments to overcome some of these restrictions include x-ray free electron lasers (XFEL) (4), which only require microcrystals, new detectors in cryo-electron microscopy (5) and in-cell NMR (6), which analyzes the structure of small proteins in a cellular context. Further advancements that assist with protein structure determination have included the development of being able to use sparse NMR data, for example using backbone only data (7), and the understanding of evolutionary constraints for predicting protein structure (8).

We present a novel approach to obtain structural details of proteins by mass spectrometry. This can be accomplished through cross-linking and mass spectrometry (CLMS) (9–11). Cross-links establish covalent bonds between residue pairs close in space but not necessarily in sequence. This conserves structural information throughout an analysis that follows the standard proteomics workflow. Typically, a bi-functional chemical reagent, the cross-linker, is incubated with a protein of interest. The cross-linker reacts with two residues—often involving the side-chain amine of lysine—that are near each other in the folded protein. A protease such as trypsin is used to degrade the protein. The resulting mix of cross-linked peptides is then analyzed by mass spectrometry and database searching akin to other shotgun proteomics approaches (12). The pairs of cross-linked residues are identified from the mass spectrometric data and provide information on which residues are near each other in the folded protein. This information is represented in the form of distance constraints, deducible from the length of the cross-linking agent.

CLMS data has been used to study large multi-protein complexes (13), networks (14) and proteins in whole cells (15). The distance constraints obtained are sparse but complement other structural data in integrated structural biology well (10). Cross-link data allow placing high-resolution structures of individual sub-units in the electron microscopy structure of an assembled multi-protein complex to obtain its quasi-atomic resolution structure, *e.g.* the proteasome (16). In an alternative approach, genetic site-directed positioning of a photo-reactive group, azide, as part of a phenylalanine analog, was recently used to derive proximity information that allowed modeling of receptor CRF1R bound to its native ligand (17). Young *et al.* used 15 cross-links to identify the correct fold of bovine basic fibroblast growth factor using threading and homology modeling (18). In a similar study, Singh *et al.* used eight cross-links to build a monomer homology model of the major capsid protein E of bacteriophage lambda and to derive a pseudoatomic model of the lambda procapsid shell (19). In both of the aforementioned cases, the cross-link information was merely used to verify structural models by threading and homology modeling, and did not significantly impact model building. Prior attempts to leverage cross-linking data in structure determination delivered improvements, however, without leading to high-resolution models (20).

Here, we increase the spatial resolution of information obtained through cross-linking by using a highly reactive chemical as a cross-linking agent. This broadens the specificity of cross-linking and thus increases the spatial resolution in conjunction with mass spectrometry. We employ the heterobifunctional chemical cross-linker sulfosuccinimidyl 4,4′-azipentanoate, sulfo-SDA (21), to chemically cross-link a protein, human serum albumin (HSA).

We combine the distance constraints provided by cross-linking and mass spectrometry with computational, conformational space search. This approach allows us to generate structural models of HSA domains that correlate highly with the structure of HSA solved by x-ray crystallography. With this method, we show that our pipeline can be used to analyze the structure of HSA domains from HSA not only in it's purified form, but additionally unpurified and in its native environment, human blood serum.

## EXPERIMENTAL PROCEDURES

### 

#### 

##### Material and Reagents

The cross-linking reagent sulfo-SDA was purchased from Thermo Scientific Pierce (Rockford, IL). Human blood serum was acquired from a healthy male donor after informed consent, in accordance with standard institutional ethical procedures at the University of Edinburgh, School of Biological Sciences. Immediately following collection (50 ml total volume split over 2× Falcon 50 ml Conical Centrifuge Tubes), blood serum was isolated from the whole blood sample without anti-coagulants, by centrifugation. Whole blood was allowed to clot by leaving it undisturbed at room temperature for 30 min. The clot was removed by centrifuging at 1900 × *g* for 10 min at 4 °C. The resulting supernatant was immediately apportioned into 1.5 ml Eppendorf Tubes as 0.5 ml aliquots, which were flash frozen using liquid nitrogen and stored in a −80 °C freezer. Protein concentration was estimated at 80 mg/ml using a Bradford protein assay.

##### Cross-Linking HSA

Mixing ratios of sulfo-SDA to HSA were titrated using cross-linker-to-protein weight-to-weight ratios of 0.25:1, 0.5:1, 1:1, 2:1, 4:1, and 8:1. Either purified HSA or whole blood serum (typically 15 μg, 0.75 mg/ml) was mixed with sulfo-SDA (typically 40 mm) in cross-linking buffer (20 mm HEPES-OH, 20 mm NaCl, 5 mm MgCl_2_, pH 7.8) to initiate incomplete lysine reaction with the sulfo-NHS ester component of the cross-linker. The diazirine group was then photo-activated using UV irradiation. A UVP B-100AP, 100 W mercury lamp at 365 nm was utilized for photo-activation. Samples were spread onto the inside of Eppendorf tube lids to form a thin film, placed on ice at a distance of 5 cm from the lamp and irradiated for either 1, 10, 20, 30, 40, 45, or 60 min. The resulting cross-linked mixture was separated on a NuPAGE 4–12% Bis-Tris gel using MES running buffer and Coomassie blue stain.

##### Sample Preparation for Mass Spectrometric Analysis

Bands corresponding to monomeric HSA were excised from the gel and the proteins reduced with 20 mm DTT, alkylated using 55 mm IAA and digested using trypsin following standard protocols (22). The resulting digests were desalted using self-made C18 StageTips (23) prior to mass spectrometric analysis.

##### Mass Spectrometry and Data Analysis

Peptides were loaded directly onto a spray emitter analytical column (75 μm inner diameter, 8 μm opening, 250 mm length; New Objectives (Woburn, MA) packed with C18 material (ReproSil-Pur C18-AQ 3 μm; Dr Maisch GmbH, Ammerbuch-Entringen, Germany) using an air pressure pump (Proxeon Biosystems) (24). Mobile phase A consisted of water and 0.1% formic acid. Mobile phase B consisted of acetonitrile and 0.1% formic acid. Peptides were loaded onto the column with 1% B at 700 nl/min flow rate and eluted at 300 nl/min flow rate with a gradient: 1 min linear increase from 1% B to 9% B; linear increase to 35% B in 169 min; 5 min increase to 85% B. Eluted peptides were sprayed directly into a hybrid linear ion trap - Orbitrap mass spectrometer (LTQ-Orbitrap Velos, Thermo Fisher Scientific). Peptides were analyzed using a “high/high” acquisition strategy, detecting at high resolution in the Orbitrap and analyzing the subsequent fragments also in the Orbitrap. Survey scan (MS) spectra were recorded in the Orbitrap at 100,000 resolution. The eight most intense signals in the survey scan for each acquisition cycle were isolated with an *m*/*z* window of 2 Th and fragmented with collision-induced dissociation (CID) in the ion trap. 1+ and 2+ ions were excluded from fragmentation. Fragmentation (MS2) spectra were acquired in the Orbitrap at 7500 resolution. Dynamic exclusion was enabled with 90 s exclusion time and repeat count equal to 1.

Mass spectrometric raw files were processed into peak lists using MaxQuant version 1.2.2.5 (25) using default parameters except the setting for “Top MS/MS peaks per 100 Da” being set to 100.

Peak lists were searched first against the human subset of UniProt (ipi.HUMAN.v3.79) using Mascot (version 2.4.0) and search parameters: MS accuracy, 6 ppm; MS/MS accuracy, 20 ppm; enzyme, trypsin; specificity, fully tryptic; allowed number of missed cleavages, two; fixed modifications, none; variable modifications, carbamidomethylation on cysteine, oxidation on methionine. This revealed that HSA was by far the most abundant protein in our bands, in both purified and human blood serum samples. Peak lists were subsequently searched against two databases using Xi (ERI, Edinburgh) for identification of cross-linked peptides. One database contained all proteins identified in the initial Mascot search, the other contained only the sequence of HSA (UniProt P02768). Search parameters were MS accuracy, 6 ppm; MS/MS accuracy, 20 ppm; enzyme, trypsin; specificity, fully tryptic; allowed number of missed cleavages, four; cross-linker, SDA; fixed modifications, none; variable modifications, carbamidomethylation on cysteine, oxidation on methionine, SDA-loop (SDA cross-link within a peptide that is also cross-linked to a separate peptide). The linkage specificity for sulfo-SDA was assumed to be at lysine, serine, threonine, tyrosine and protein N termini at one end, with the other end having specificity for any amino acid residue. False discovery rates (FDR) were estimated following a modified target-decoy search strategy (22, 26). In both searches, only cross-links in HSA were identified and we henceforth only consider the results of the search done against the HSA sequence alone. The MS data have been deposited to the ProteomeXchangeConsortium via the PRIDE partner repository with the data set identifier PXD001692 (27). Cross-link results from the FDR analysis with scores at the peptide-spectrum match level and confidence values at cross-link level are also available in supplemental Tables S1–S4. Cross-links were visualized in the crystal structure of HSA (PDB 1AO6) (28) using PyMOL (29).

##### Domain Boundary Prediction in Conformational Space Search

The full-length structure of HSA (PDB 1AO6) is comprised of 576 residues. Proteins of this size are challenging for conformational space search as the search space grows exponentially with protein length. To demonstrate the feasibility of our combined CLMS/search approach, we decided to split HSA into smaller domains more suitable for state-of-the-art protocols. We employed the following computational domain boundary prediction methods: DoBo (30), Threadom (31) and the domain boundary module from the PSIPRED (32) server. We used the average scores from all servers to obtain a domain boundary prediction. Individual predictions and the consensus are given in supplemental Table S5.

##### Conformational Space Search with Realistic Energy Functions by Model-based Search

We performed conformational space search based on cross-linking/mass spectrometry (CLMS) constraints using a modification of our previously described model-based search (MBS) (33). Model-based search is integrated into the Rosetta Modeling framework (34) and uses Rosetta's low-level algorithmic foundation. Rosetta assembles structural fragments from a library to sample protein structures. The fragment library is derived from sequence profiles and secondary structure prediction. Conformational space search combines a low-resolution and a high-resolution stage. During the low-resolution stage, side-chains are modeled as centroid pseudo-atom. In the subsequent high-resolution stage, Rosetta employs a realistic, hybrid all-atom physical/knowledge-based force field to refine protein structures. The version of MBS described in this article is integrated into the release version of Rosetta 3.4. For all our prediction experiments, we use homology-free fragment libraries.

MBS operates by generating a set of candidate structures based on a small number of Rosetta's Monte-Carlo runs. The resulting structures are clustered by a heuristic clustering procedure to identify densely sampled low-energy regions. We interpret these regions to represent funnels in the energy landscape. MBS then judges the quality of a funnel by refining the five lowest-energy structures in an all-atom force field. The different funnels, along with their estimated quality, form an approximate model of the energy landscape. In subsequent iterations, MBS reallocates computational resources from low-quality funnels to promising regions of the conformational space.

MBS consists of six stages. Stage 1 builds coarse topologies, using stages 1 and 2 of Rosetta's *AbInitioRelax* protocol. In the following 4 MBS stages, the conformational space is searched by 9-mer fragment replacements (Rosetta stage 3). The resulting structures are refined in the final MBS stage by 3-mer fragment replacements (Rosetta stage 4). Each stage of MBS is interleaved with a clustering step to determine an approximate model of the energy landscape and to reallocate computational resources, as described above. We generate 5000 structures in each stage of MBS.

The three HSA domains contain long loops and we accounted for their high flexibility in modeling by the following treatment: We explicitly modeled the long loop regions (longer than 15 amino acids) predicted by DISOPRED2 (35), but removed them from scoring by only considering the repulsive terms of the loop regions, as described by Wang *et al.* (36). The same procedure is applied to N/C-terminal residues that are predicted to be disordered by DISOPRED2. This results to the following residues that are considered for full scoring in the all-atom phase: 2–71:115–194 for domain A, 200–262:308–381 for domain B and to 389–458:508–571 for domain C. The RMSD reported in the manuscript is calculated over these residues.

##### Modeling of Cross-link Constraints

For CLMS constraints obtained by SDA, we used 20Å as the upper bound for the distance between solvent-accessible amino acids. We set the upper bound to 20Å, the maximum through-space Cα-Cα distance of amino acids with long side-chains (like lysine and arginine) plus the sulfo-SDA linker distance and some added distance to allow for conformational flexibility. This is a conservative estimation of the upper distance bound of the SDA cross-linker and agrees with the experimentally observed upper limits when comparing CLMS data with the crystal structure of HSA (see Fig. 2*C*). To model these constraints as part of the energy function during conformational space search, we use a modified Lorentz function:

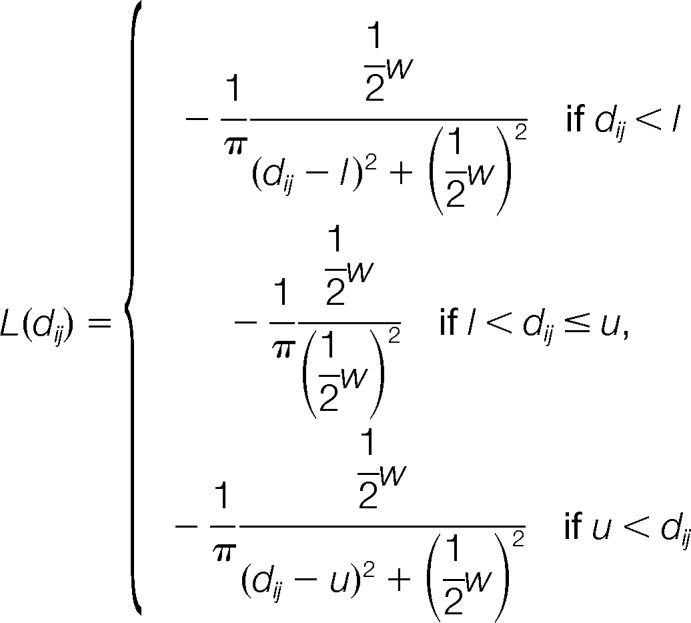
 where *d_ij_* is the distance between residues *i* and *j* in the structure, the lower bound *l* = 1.5Å and the upper bound *u* = 20Å are distance limits within which the maximum energy bonus (*E_max_*) is rewarded. The parameter *w* = 1.0 regulates how quickly the energy bonus decreases when *d*_ij_ is not within the lower/upper bounds, with *w* being the half-width *i.e.* the violation in Å where *E_max_*/2 is still rewarded.

If a constraint is satisfied (*l*<*d*_ij_≤*u*), the Lorentz term will award an energy bonus E_max_. If the constraint is mildly violated *d*_ij_-*l* ≤ 2*w* or *u-d*_ij_ ≤ 2*w*, the energy bonus decreases. However, if the constraint is significantly violated (*l-d*_ij_>2*w* or d_ij_-*u*>2*w*), the influence of the constraint will approach zero, effectively ignoring the constraint in the energy calculation. Intuitively, the resulting energy term attempts to maximize the number of constraint satisfied by a structure, rather than the exact distances.

##### Contact Prediction

To augment the structural information contained in CLMS constraints, we perform contact prediction using our novel contact prediction algorithm. This algorithm, EPC-map, predicts contacts based on evolutionary information and physicochemical information in structure models obtained using *de novo* prediction methods (37). It generates 1000 Rosetta models of the target protein. Contacts in resulting structures are scored by a support vector machine trained to predict native contacts based on the local, physicochemical context of a contact and by several sequence-based features, such as residue conservation obtained from multiple-sequence alignments. Contact predictions are made by combining the weighted sum of scores from the support-vector machine, consensus over the top 2% of predicted structures, and prediction scores from PSICOV (38), which predicts contacts from multiple-sequence alignments by sparse inverse covariance estimation. Note that the results in this article have been generated with an early version of EPC-map that uses PSICOV for estimating evolutionary contacts. PSICOV has been replaced by GREMLIN (39) in the current version of EPC-map (37).

##### Application of CLMS Constraints and Predicted Contacts in Model-based Search

We employ CLMS constraints together with predicted contacts to guide search during all low-resolution stages of MBS. We use CLMS constraints at 20% FDR. We predict the structure of the individual domains of HSA (PDB 1AO6) using MBS and 320/107/248 CLMS distance constraints for domains A/B/C for purified HSA in solution and 248/68/163 CLMS constraints for HSA in blood serum. Note that we only used CLMS constraints with a sequence separation larger than 12aa. These longer constraints are more informative than short-range constraints. The latter mainly carry spatial information from secondary structure elements.

The resulting algorithm for structure determination combines experimental information from CLMS constraints with physicochemical information captured in the energy function (from contacts as well as through the use of MBS). This combined information enables effective conformational space search. Our results serve as a preliminary indication that the synergistic combination of experiment and computation may lead to efficient, cost-effective, high-throughput structure determination methods.

##### Structure Selection

We tested several methods for structure selection in their ability to select a single structure (first structure), and a low-RMSD structure within a small number (we choose five in this study) of top ranked structures (best structure). We tested Rosetta's physically realistic all-atom energy function, clustering with Durandal (40), Lorentz energy of satisfied CLMS constraints, a knowledge-based potential (ProSA) (41), and an orientation-dependent statistical potential (GOAP) (42) for structure selection. Structures are selected from all funnels across all stages of MBS. We also find that the low-energy ensemble of MBS usually contains low-RMSD structures. Thus, we tested some combinations of Rosetta energy and the methods (Rosetta energy+CLMS constraints and Rosetta energy+GOAP) in their ability to select structures with native-like backbone combinations. For the method combinations, we consider the ten structures with lowest Rosetta energy (coarse first filtering) and re-rank them with the second method (CLMS constraints or GOAP).

## RESULTS AND DISCUSSION

### 

#### 

##### CLMS of purified HSA and human blood serum

We hypothesize that CLMS data contains sufficient information to define the structure of HSA in solution. This may require more data, however, than obtained by the currently used highly specific reagents. To increase the density of cross-linking data, we need to increase the number of cross-links that we are able to produce and identify. We achieve this using sulfo-SDA because it is less specific in it's reactivity than standard homobifunctional NHS-ester based cross-linkers. On one side sulfo-SDA carries a traditional NHS-ester that reacts with protein N termini and the side chains of lysines as well as to a lesser extent those of serine, threonine and tyrosine. On the other end the cross-linker has a UV photoactivatable diazirine (supplemental Fig. S1). This group is stable under normal conditions but can be activated by light (320–370 nm) to generate a highly reactive carbene (43). Carbenes have vanishingly short half-lives in water and react within femtoseconds with organic molecules (44). One would therefore expect SDA cross-links to form between K/S/T/Y on one side and any amino acid on the other.

There are numerous factors that make HSA the model system of choice in this study: in cross-linking terms it is a medium sized protein (∼66 kDa), it has a known crystal structure and is commercially available at a low cost in a purified form. HSA is also the most abundant protein in human blood plasma, making up more than half the total protein content. One of the technical challenges for CLMS is the mass spectrometric detection of cross-links, which is easier in low rather than high complex mixtures. Enriching for the target protein following the cross-linking step reduces mixture complexity and enhances cross-link detection. Also other structure determination approaches, NMR and x-ray crystallography, require protein purification. Notably, the purification requirements are large for these established approaches with regards to protein amount and purity. Furthermore, they require the protein to be native throughout the enrichment as the structure is subsequently analyzed. CLMS fixes the structure first, allowing the enrichment to employ procedures that would otherwise disrupt protein structure, *e.g.* SDS-PAGE. Indeed, the abundance of HSA in blood serum allows us to access sufficiently pure protein through use of SDS-PAGE alone.

**Fig. 1.**
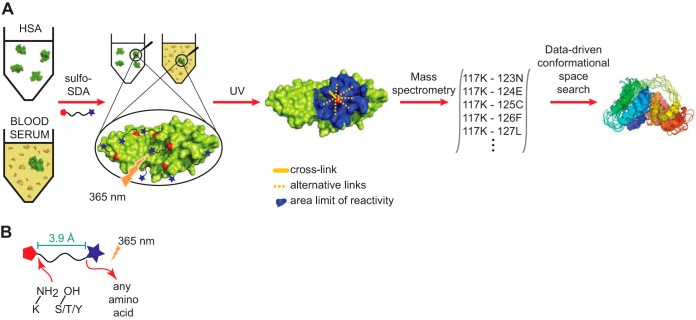
**Workflow of photo-cross-linking/mass spectrometry combined with computational conformational space search.** *A*, purified HSA and human blood serum were cross-linked using photo-reactive sulfo-SDA in a two-step procedure. Proteins are first decorated by the cross-linker at Lys, Ser, Thr, Tyr and N terminus. Upon UV activation, the cross-linker links these residues to a nearby residue. The cross-linked protein is then subjected to a proteomic workflow, consisting of trypsin-digestion, liquid chromatography-mass spectrometry and database searching to identify the cross-linked residues. These intramolecular proximities are then used as experimental constraints during computational conformational space search. *B*, schematic view of the cross-linker.

**Fig. 2.**
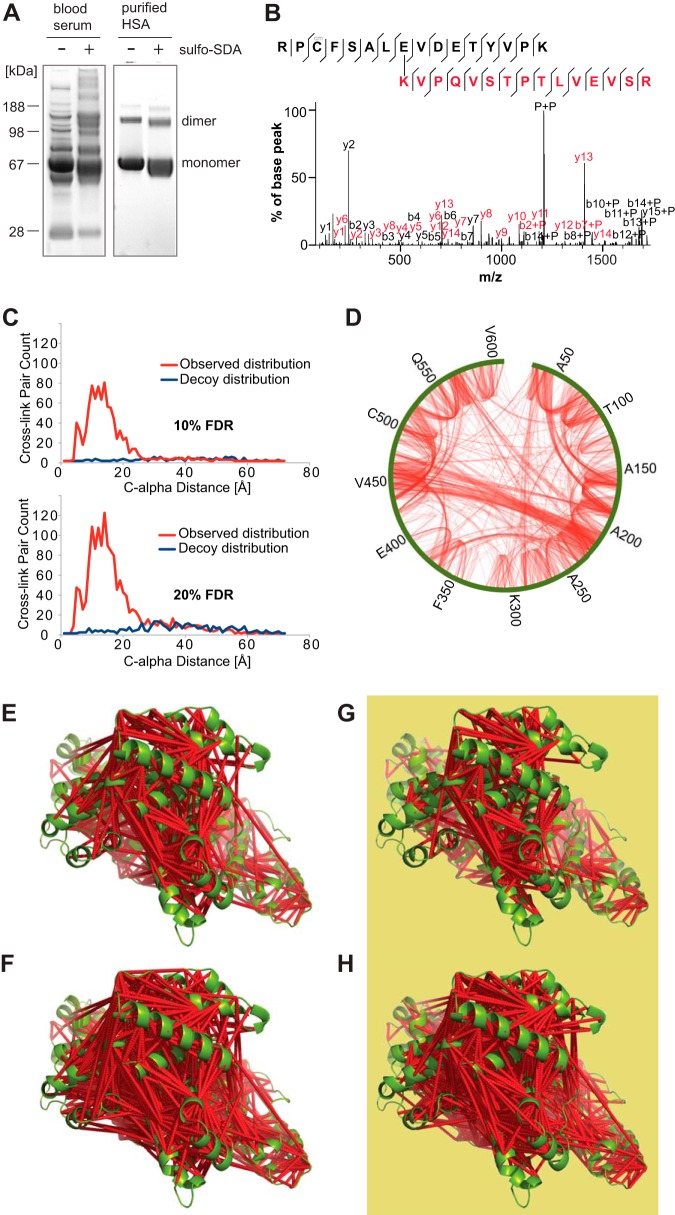
**Purified HSA and HSA in blood serum and mass spectrometry.** *A*, blood serum proteins and purified HSA, with (+) and without (-) sulfo-SDA cross-linking. *B*, high-resolution fragmentation spectrum of SDA cross-linked peptides that reveals the intramolecular proximity of K437 and E492. *C*, FDR analysis showing observed distance distribution in comparison to the decoy distance distribution. The plot shows the residue-residue C-alpha distances of cross-linked residues and decoy database hits as observed in the crystal structure PDB 1AO6 for the respective residue pairs. *D*, cross-link network (*n* = 1,495, 20% FDR) for purified HSA. Green outer line represents the sequence of HSA. *E* and *F*, cross-linked residue pairs of purified HSA in PDB 1AO6: *E*, *n* = 881, 10% FDR; *F*, *n* = 1,495, 20% FDR. *G* and *H*, cross-linked residue pairs of blood serum HSA in PDB 1AO6: *G*, *n* = 644, 10% FDR; *H*, *n* = 1,304, 20% FDR.

We cross-linked purified HSA and human blood serum using sulfo-SDA in a two-step procedure. First, protein was labeled by sulfo-SDA in the dark and then the labeled protein was exposed to UV light. The cross-linked protein was subjected to PAGE, HSA excised and subsequently digested and analyzed by LC-MS using a high-high acquisition strategy (Fig. 1, see “Experimental Procedures”). The reduced selectivity resulting from the presence of the photo-reactive diazirine on sulfo-SDA, increases the number of observed distance constraints significantly (Fig. 2). Using the highly selective standard cross-linker Bis(sulfosuccinimidyl)suberate (BS3), 43 distance constraints for HSA had been previously reported (45). In contrast, using sulfo-SDA, we obtained 205/500/881/1495 at 1/5/10/20% false discovery rate (FDR) for purified HSA. This was the result of 87 acquisitions, each of an estimated 5 μg HSA, from a starting 15 μg cross-linked HSA from the SDS-gel. No optimization was done to minimize the number of acquisitions at this point. Each acquisition added further identifications, although half of the residue pairs (20% FDR) were identified by any random subset of 13.2 ± 1.6 runs (supplemental Fig. S2*A*). Proteomics generally suffers from stochastic detection of analytes especially when these are present at low levels. This is not unique to cross-links but also affects linear peptides albeit to a much lesser extent owing to their generally higher intensity (supplemental Fig. S2*C*). For linear peptides a single run essentially returns 50% of the identifications under our conditions that focused on cross-linked peptides (singly and doubly charged precursors excluded). As we discuss later, the high number of constraints at 10 and 20% FDR has larger value for protein structure modeling than the increased confidence at lower FDR. Thus we focus our results discussion on CLMS data at these FDR rates (Fig. 2). We identified 644/1304 distance constraints at 10/20% FDR in blood serum HSA. This was the result of 117 acquisitions, each of an estimated 5 μg HSA, from a starting 15 μg cross-linked human blood serum loaded on the SDS-gel. As for purified HSA, each acquisition added further identifications, with half of the residue pairs (20% FDR) identified by any random subset of 11.5 ± 2 runs (supplemental Fig. S2*B*, S2*D*). The majority of the observed constraints in blood serum HSA are in agreement with those of purified HSA (supplemental Fig. S3). supplemental Tables S1 and S3 contain the peptide-spectrum matches and supplemental Tables S2 and S4 contain all cross-links from this work for up to 20% FDR, along with a confidence score.

A cross-linked peptide comprises two peptides and a linkage site within each. NHS-esters have limited reactivity, which helps in assigning linkage sites. The total promiscuity of the carbenes resulting from photoactivated diazirine means that the diazirine cross-linking end can theoretically be placed at any residue within a peptide. For accurate site assignment one requires backbone fragmentation on either side of a linkage site. We manually checked for the supporting evidence of the linkage sites returned by our search, for a random subset of 78 unique cross-links and 368 supporting spectra with wide score distribution. We found that in 38/78 (49%) of the unique cross-links and 77/368 (21%) of matching spectra, the diazirine linkage site was supported by at least one fragmentation event on either side of the assigned site. In cases where there is no fragmentation evidence to pinpoint the exact site of diazirine reaction, our algorithm places the linkage site on the first amino acid within the region indicated by flanking fragmentation events. Some of these sites will be wrong and future implementations should report supported regions rather than imprecise sites. Site imprecision in addition to false sites are therefore errors that need to be considered during protein modeling.

We gave consideration to testing the possible impact of imprecise site calling on our constraint data quality. For this, we compared our distance distribution of linked residue pairs with a partially randomized data set (supplemental Fig. S4). The randomization was done by locally shifting all (diazirine) sites randomly once within a window of residues. We considered window sizes from 3 to 39 residues and all residue pairs that served as input in our protein modeling (residue pairs more than 12 residues apart in the protein sequence, 20% FDR, see below). Assuming *p* = 0.05 to be the significance threshold, distance distributions differed significantly only when considering a window of 11 residues. This means that our site assignment could have erred within ± 5 residues around our called site before the resulting distance distributions would have been significantly different. However, this does not mean that at this point the modeling would have been affected. The median shift of the distribution is very small; at window size 11 the distribution shifts by 1.00 ± 0.16 Å and even at window size 39 it does not shift more than 4 Å. Note that the median length of our diazirine cross-linked peptides is 12 residues. Consequently, identifying the peptide suffices and calling a random residue within this peptide as linkage site hardly impacts on the distance distribution.

**Fig. 3.**
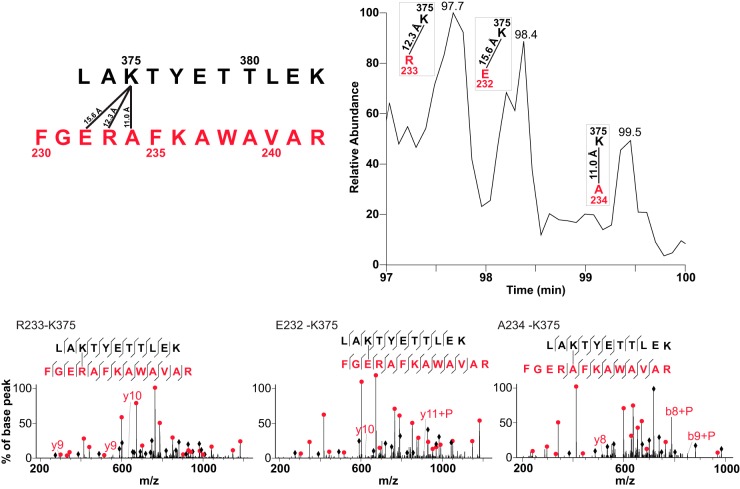
**Extracted ion chromatogram of a cross-linked peptide pair.** Cross-linked peptide pair labeled with sequence number with first peptide match shown in *black* and second peptide match in *red*. K375 found cross-linked to three residues (R233, E232, and A234) in the second peptide in a single LC-MS run. Peaks in the extracted ion chromatogram (97.67, 98.38 and 99.45 mins) are labeled with the sites of cross-linking in the peptide pair matched by the database search software, Xi. C-alpha distances are indicated on cross-linked residue pairs. Fragmentation spectra for each cross-linked peptide pair are shown at the bottom as evidence of identification. Fully annotated spectra are provided in supplemental Fig. 10.

We made two noteworthy observations regarding linkage sites in our data. Identical peptide pairs with different linkage sites are separated by liquid chromatography during our analysis (Fig. 3, see supplement for fully annotated spectra). We looked at cross-links between a pair of identical peptides, involving a lysine residue (K375) on peptide one and three residues (E232, R233 and A234) on peptide two. The three different cross-linking sites were identified in a single LC-MS run. Matching the cross-linked peptide spectrum scan numbers to the raw data file of this run revealed three distinct peaks on the LC-MS chromatogram, each corresponding to a different cross-link position on the second peptide.

**Fig. 4.**
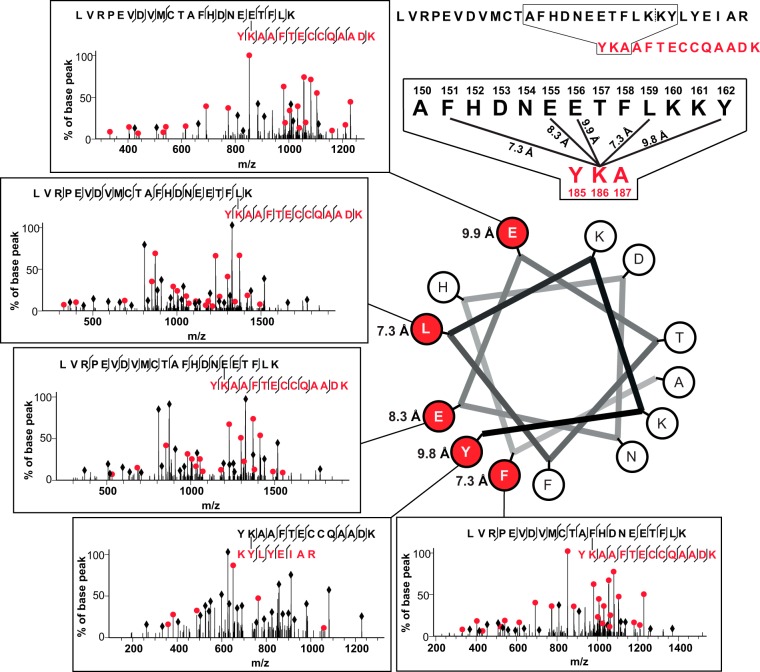
**Identified cross-linked sites suggest alpha-helical secondary structure.** K186 from peptide two (sequence shown in *red*) found cross-linked to five residues (F151, E155, E156, L159 and Y162) from peptide one (sequence shown in *black*). The full sequences of peptides identified as cross-linked peptide pairs are shown at the top right, with an expansion of the sequence showing residues cross-linked to K186 shown underneath. Residues from the sequence of peptide one are shown assembled in a representation of an alpha-helix, beginning with A150 and ending with Y162. Residues identified as cross-linked to K186 are colored *red*, with the associated fragmentation spectra for each cross-linked peptide pair shown as evidence of identification. C-alpha distances are associated with each cross-linked residue pair. Fully annotated spectra are provided in supplemental Fig. 11.

A further feature of sulfo-SDA is the level of detail that can be described by the resulting identified cross-links. In one instance, where we performed a manual validation on the placement of the diazirine reactive site, we found that we were able to elucidate evidence of protein secondary structure based on the pattern of amino acid cross-linking sites that we identify (Fig. 4). A lysine residue (K186) on one peptide was found cross-linked to five other residues over a range of 12 amino acids, with cross-links occurring at F151, E155, E156, L159 and Y162. This pattern is a curiosity until the amino acid sequence of the second peptide is assembled as an alpha helical wheel, beginning with A150 and ending with Y162. When this was carried out, it became clear that the observed pattern is suggestive of lysine residue K186 cross-linking with the available amino acid residues on an alpha helix in close proximity. This could be confirmed upon examination of the x-ray crystal structure for HSA.

##### Conformational Space Search to Determine HSA Domain Structures

For the 585 residues of HSA and given the number of cross-links identified, it means that on average, we determined an average of 1.5/2.5 constraints per residue at 10/20% FDR. This is approaching the frequency obtained in NMR spectroscopy (3–20 constraints per residue) (46), albeit for a protein of 66 kDa, a substantially larger size than typically investigated by NMR. However, the number of constraints per residue obtained with CLMS remains at this point insufficient for structure determination with standard NMR protocols (data not shown). Nevertheless, we hypothesized that our increase in distance constraints obtained by CLMS passes a critical threshold: the CLMS data from our experiment does indeed contain sufficient information to reconstruct the domain structures of human serum albumin. To demonstrate this, we combine the information from CLMS with the information encoded in the energy potentials of state-of-the-art *de novo* structure prediction algorithms. We use an algorithm called model-based search to integrate CLMS data with conformational space search for structure modeling (Fig. 1 for an outline of the method and “Experimental Procedures” for details) (33). Earlier studies used cross-link data to build monomer structure models by threading and homology modeling (18, 19), which requires homologous template structures in the Protein Data Bank. However, in both studies the cross-link information was used for model verification and did not significantly impact the building of monomer structure models. In contrast, our approach uses a much larger number of cross-links to build and verify protein structure with a *de novo* structure prediction algorithm. Thus, the procedure is applicable to proteins without structural homologues that are required for homology modeling.

Typically, searching the conformational space for the native structure is difficult because of the size of the space and the ruggedness of the energy landscape, although for small proteins this has been achieved (47). However, the structural information contained in CLMS constraints directs search toward near-native conformations, such that sampling of lower root-mean-square deviation (RMSD) structures is greatly increased. To direct the search, we integrate CLMS distance constraints into the energy function with a Lorentzian function, deepening valleys in the low-resolution energy landscape where distance constraints are approximately satisfied (details are provided in the “Experimental Procedures” section). In the modified landscape, search for low-energy regions becomes much more effective. At the same time, we designed our algorithm to cope with the inherent noise in CLMS data. The Lorentzian function deals with noise by maximizing the number of satisfied constraints, rather than penalizing constraints that are not satisfied.

**Fig. 5.**
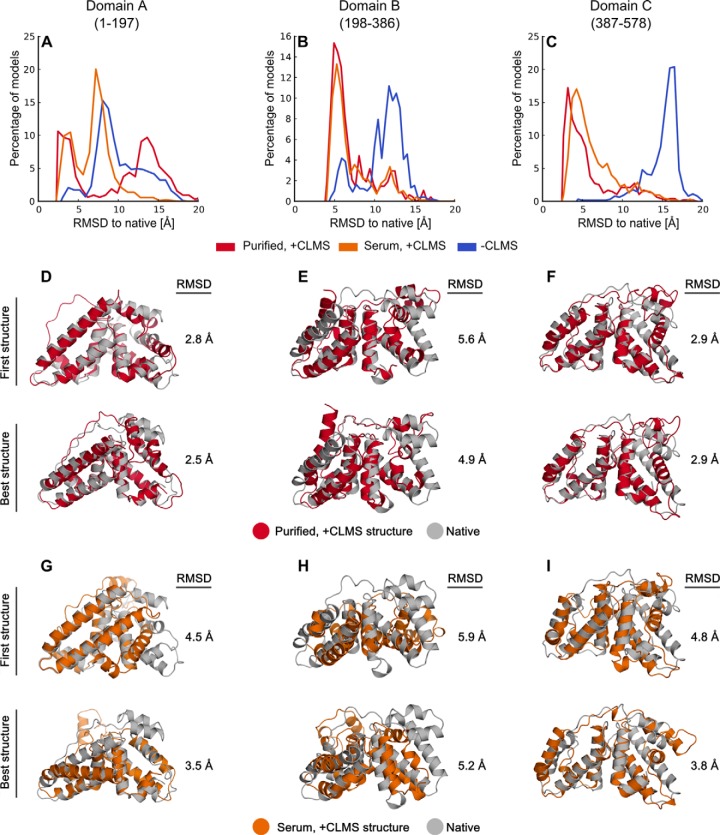
**Determined structures for the individual domains of HSA by using cross-link constraints and conformational space search.** *A–C*, deviation of domain structures obtained with our novel procedure to the crystal structure of HSA (PDB 1AO6). CLMS data from purified and serum HSA (red and orange curve) increases the sampling of low-RMSD structures, compared with structures obtained without CLMS data (blue curve). *D-F*, first and best determined structures calculated with CLMS data from purified HSA, aligned to the crystal structures of the HSA domains. For each domain, “First structure” refers to the single structure we selected using Rosetta energy+CLMS constraints. “Best structure” refers to the lowest RMSD structure to PDB 1AO6 among the best five structures ranked by Rosetta energy+GOAP (see “Experimental Procedures”). *G–I*, first and best determined structures calculated with CLMS data from HSA samples in serum. Large loops in the crystal structure and terminal residues predicted to be disordered are removed for RMSD computation (see “Experimental Procedures”). The following residues are used for calculation of the RMSD: 2–71:115–194 for domain A, 200–262:308–381 for domain B and 389–458:508–571 for domain C.

Using this procedure, we generated structures of the three domains of HSA using the CLMS constraints at 20% FDR from purified HSA and from HSA samples in serum. The division of HSA into domains is necessary, as existing computational methods cannot yet address the size of proteins that can be analyzed experimentally (see “Experimental Procedures” and supplemental Table S5). Domains of purified HSA have the following sizes and number of CLMS constraints: domain A: 197 amino acids, 22.6 kDa, 320 CLMS distance constraints; domain B: 189 amino acids, 21.5 kDa, 107 distance constraints; domain C: 192 amino acids, 21.7 kDa, 248 distance constraints, we only used CLMS constraints with a sequence separation of 12 or more residues. CLMS data leads to increased sampling of near-native conformations compared with sampling without constraints (Fig. 5*A*–5*C*). The structures determined for domains A/B/C of purified HSA have an RMSD of 2.8/5.6/2.9 Å to the crystal structure (PDB 1AO6) (Fig. 5*D*–5*F*, supplemental Table S6). We mostly find deviations from the crystal structure in the long loop region, probably because of its inherent flexibility, and in the interface regions between domains. However, the low energy ensembles display good convergence and sampling around the native structure (supplemental Fig. S5). The relatively high RMSD of the model of domain B is the result of a reduced number of available CLMS distance constraints for this domain.

Without the use of CLMS constraints, the RMSDs for the best models obtainable by conformational space search alone have low resolution (RMSD 7.9/7.4/15.5 Å for the best structure among the ten lowest-energy structures) (Fig. 3, see also supplemental Table S6). In addition, we tested the impact of BS3 cross-linking data on structure modeling success and found BS3 information insufficient to aid the modeling process (RMSD 10.2/11.9/10.4 Å for the best structure among the ten lowest-energy structures) (supplemental Fig. S6 and supplemental Table S6).

We also tested the impact of the FDR on the backbone quality of the structure ensemble to investigate the tradeoff between cross-link accuracy and quantity. We repeated the MBS calculations five times with CLMS data at 1/5/10/20% FDR and quantified the ensemble quality by the RMSD at the 1% percentile (supplemental Fig. S7). Overall, MBS samples the lowest RMSD structures with CLMS data at 10 or 20% FDR. Except for domain A (purified), 20% FDR yields the highest ensemble quality, with the RMSD at the 1% percentile dropping from 4.1/5.3/6.4 Å (1% FDR) to 3.6/4.2/3.1 Å (20% FDR), for domains A/B/C. Thus, a high number of CLMS constraints, even at significant degrees of noise (10–20%), is more effective in modeling HSA domains than few, accurate links at low FDR (1–5%). This is especially true for domain C: The average backbone quality of the sampled structures improves dramatically from 6.4 at 1% FDR to 3.1 Å at 20% FDR.

Furthermore, we tested several methods to select structures out of the structure ensemble (see “Experimental Procedures” and supplemental Fig. S8). The combination of Rosetta Energy and CLMS constraints is able to consistently select a single structure with correct topology (RMSD smaller than 6 Å) from the structure ensemble (denoted as “First structure” in Fig. 5). This procedure selects structures with good agreement to the native structure for purified HSA domains (2.8/5.6/2.9 Å for domains A/B/C). However, the first selected structure might not necessarily be the best because of inaccuracies in the energy function and noise in CLMS data. Thus, we also assessed the ability of structure selection methods to rank low-RMSD structures within a low number of structures that can be considered for manual inspection (we choose five structures in this work because this number is typically considered by the structure modeling community, supplemental Fig. S9). Rosetta energy in combination with an orientation-dependent all-atom statistical potential (GOAP) performs best for HSA domains (2.5/4.9/2.9 Å for domains A/B/C). The best-out-of- five structures selected by Rosetta energy+GOAP are closer to native than the first structures selected by Rosetta energy+CLMS constraints (Fig. 5). Thus, we recommend manual inspection of the top five structures, especially if additional (experimental) data and/or biological knowledge is available and can be leveraged for structure selection.

We repeated the modeling experiments on the HSA domains with CLMS data from serum samples to test the ability of the approach to probe the structure of HSA in its native, biological environment. Once cross-linking has taken place, a protein can be isolated without concerns about its structural integrity. We tested this by cross-linking HSA in serum and then enriching HSA by running SDS-PAGE (Fig. 2). The RMSD distributions for HSA CLMS in-serum structures and purified CLMS HSA structures show significant overlap, demonstrating that CLMS information acquired by starting with HSA in serum has great utility for structure determination. For domains A and C, the first generated structures are in agreement with the native structure, RMSD 4.5 and 4.8 Å, respectively (Fig. 5*G*–5*I*). The structure of domain B shows larger deviations from native (RMSD 5.9 Å), because of a smaller number of CLMS constraints (248/68/163 constraints for domains A/B/C) (supplemental Tables S2 and S4), but still coarsely resembles native topology. The best of five structures are in close agreement with the native structure (RMSD 3.5/5.2/3.8 Å for domains A/B/C, Fig. 5*G*–5*I*).

Interestingly, slightly better first structures can be selected with CLMS data from purified HSA than with CLMS data from serum samples (RMSD improvement from (4.5/5.9/4.8 to 3.6/5.9/4.5 Å). Although this would not be possible in a real application in complex environments, this is another indication that CLMS data is valuable for conformational space search and structure selection.

## CONCLUSIONS

We presented an approach that combines experimental high density data from photo-CLMS with conformational space search to recapitulate the structure of human serum albumin (HSA) domains in solution. We also showed that by combining high density data from photo-CLMS with computational biology we are able to study the structure of HSA domains in a complex mixture of proteins, human blood serum. HSA is the most abundant protein of human blood serum, a fact that largely simplified the enrichment of the protein after the cross-linking reaction. Less abundant proteins will require a more elaborate enrichment than simply running an SDS-PAGE, as was done here. However, our proof-of-concept success with HSA suggests the possibility that photo-CLMS and computational biology will reveal the structure of other proteins in their native environment. Prior to routine application, our approach now needs to be optimized and improved in many directions including chemistry, sample preparation, mass spectrometric acquisition, reducing the number of required acquisitions, data analysis and use of constraints during modeling. Ultimately, we envision photo-CLMS and conformational space search will be an experimentally simple and cost-effective complement to established structure determination methods, NMR and x-ray crystallography.

## Supplementary Material

Supplemental Data

## Footnotes

^1^ The abbreviations used are:

NMR: nuclear magnetic resonance
sulfo-SDA: sulfosuccinimidyl 4,4′-azipentanoate
NHS: *N*-hydroxysuccinimide
XFEL: X-ray free electron lasers
CLMS: cross-linking/mass spectrometry
HSA: human serum albumin
IAA: iodoacetamide
LTQ: linear trap quadrupole
MS2: tandem MS scan
MBS: model-based search
LC-MS: liquid chromatography mass spectrometry
RMSD: root-mean-square deviation.
