# Supplementary material for: Serum Albumin Domain Structures in Human Blood Serum by Mass Spectrometry and Computational Biology
Source: Mol Cell Proteomics. 2015 Sep 18;15(3):1105–16. doi: 10.1074/mcp.M115.048504 (PMC4813692; doi:10.1074/mcp.M115.048504)
Supplement: Supplemental Data [file supp_15_3_1105__index.html]

Serum Albumin Domain Structures in Human Blood Serum by Mass Spectrometry and Computational Biology — Serum Albumin Domain Structures in Human Blood Serum by Mass Spectrometry and Computational Biology — Protein Structure Determination in Biological Matrices — Supplemental Data 

# Serum Albumin Domain Structures in Human Blood Serum by Mass Spectrometry and Computational Biology

## Supplemental Data

- Supplemental Materials (.pdf, 2.0 MB) - Supplemental Materials for Main Manuscript
- Supplemental tables 1-4 (.zip, 2.9 MB) - Supplemental tables 1-4
