# Supplementary material for: Serum Albumin Domain Structures in Human Blood Serum by Mass Spectrometry and Computational Biology
Source: Mol Cell Proteomics. 2015 Sep 18;15(3):1105–16. doi: 10.1074/mcp.M115.048504 (PMC4813692; doi:10.1074/mcp.M115.048504)
Supplement: Supplemental Data [file 10.1074_M115.048504_mcp.M115.048504-1.pdf]

Supplemental Materials for:

Human Serum Albumin Domain Structures in Serum by Mass Spectrometry and Computational Biology

A. Belsom, M. Schneider, L. Fischer, O. Brock, J. Rappsilber

Supplemental Figures 1-9

Supplemental Tables 1-6

Supplemental Figures:

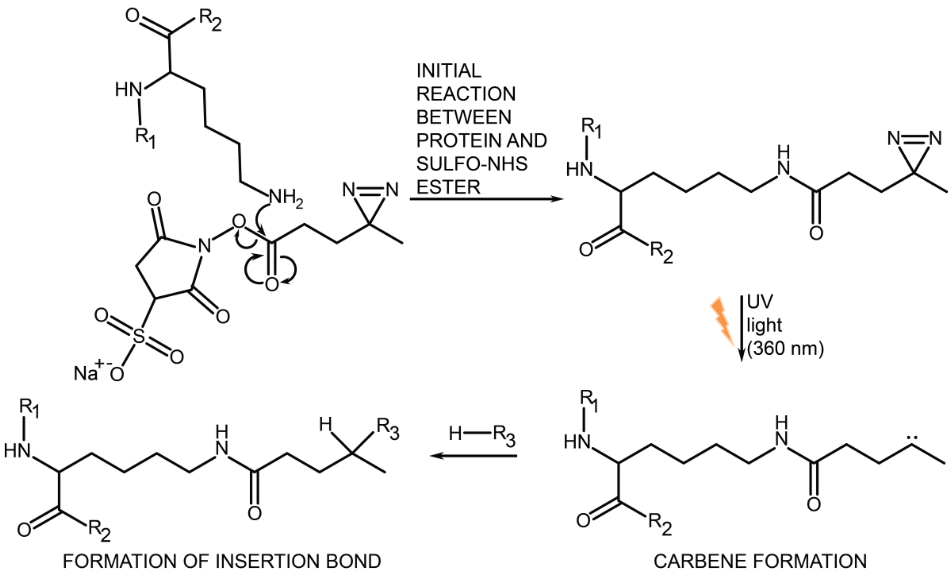

Supplemental Figure 1. Sulfo-SDA reactivity. Reaction scheme for sulfo-SDA.

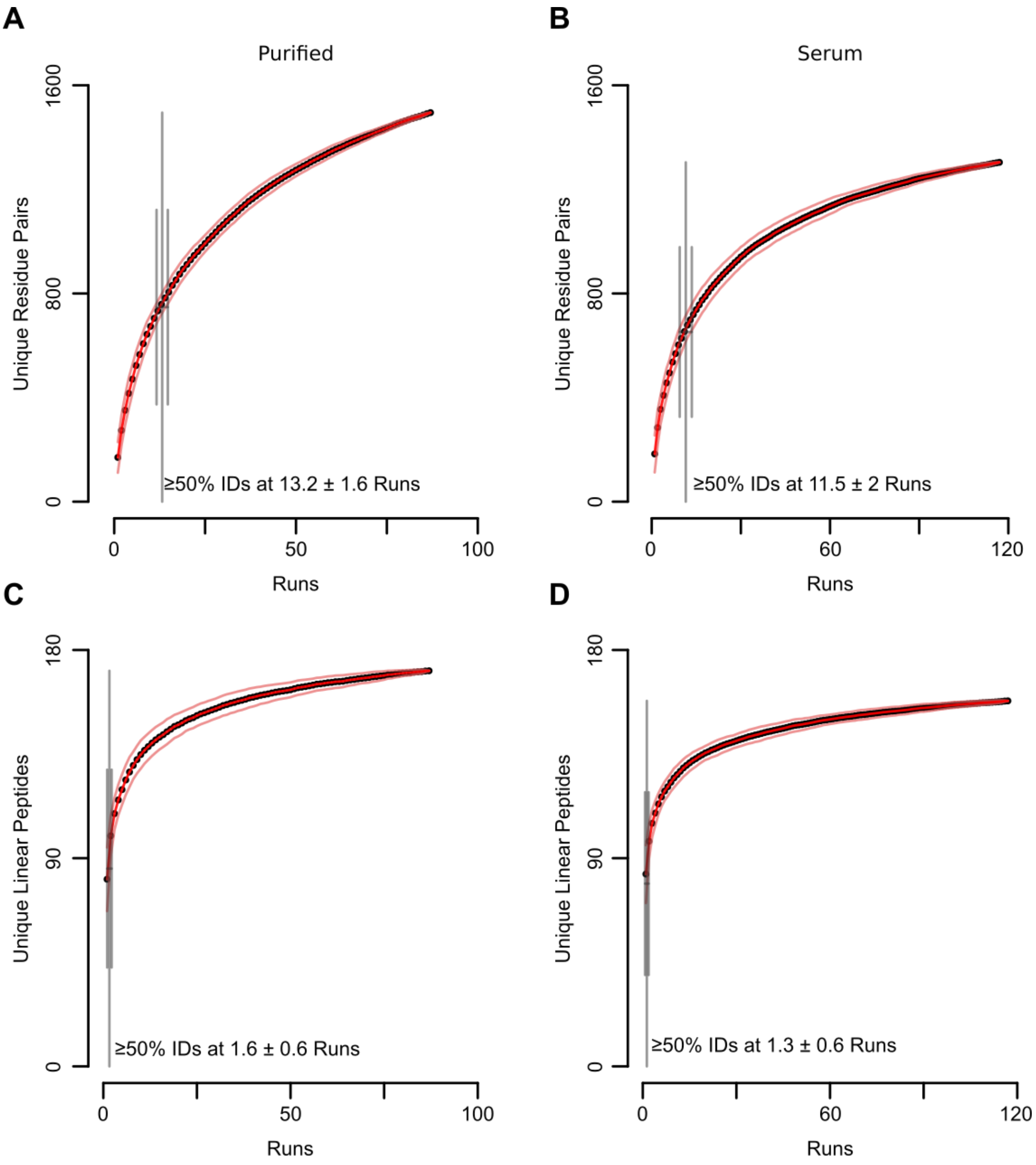

Supplemental Figure 2. Residue pair and linear peptide identifications accumulated over runs. A and **B**: Total number of unique residue pairs (20% FDR) increases with each successive LC-MS run for cross-linked purified HSA (A) and cross-linked blood serum HSA (B). **C** and **D**: Total number of linear peptides identified (5% FDR) in the same raw data as used in A and B increases with each successive LC-MS run for cross-linked purified HSA (C) and cross-linked blood serum HSA (D). The order of LC-MS runs in the series was permutated 100 times and the mean increase per run in all permutations is plotted. The standard deviation for each point is plotted as error bars. Four missed cleavages were allowed and only unique residue pairs or peptide sequences, respectively, were counted (i.e. modifications were ignored during the counting of unique IDs). This allowed for linear peptides the maximum possible number of peptides (sequence of HSA, requiring at least 7 residues, allowing for up to 4 missed cleavages) to be predicted at 335. This means that still not all theoretically possible peptides were seen, keeping in mind that trypsin missing more than one cleavage site is a very rare event and thus such peptides are seen with very low intensities. Acquiring additional runs creates more opportunity to identify peptides that are observed with signals near the detection limit.

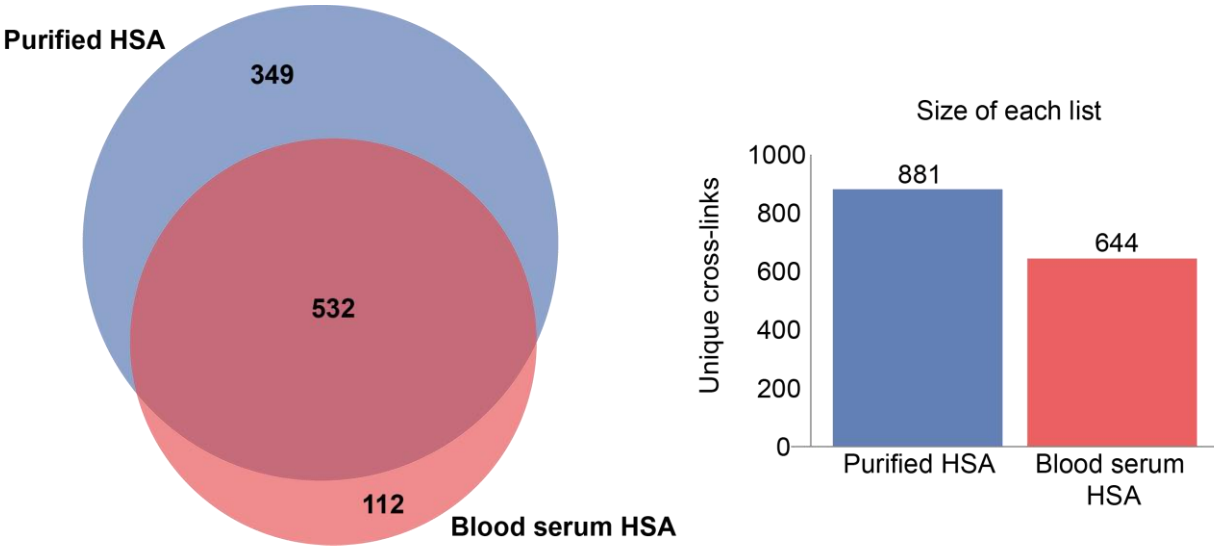

Supplemental Figure 3. Venn Diagram showing the overlap of CLMS constraints derived from purified HSA and from HSA in blood serum at 10% false discovery rate.

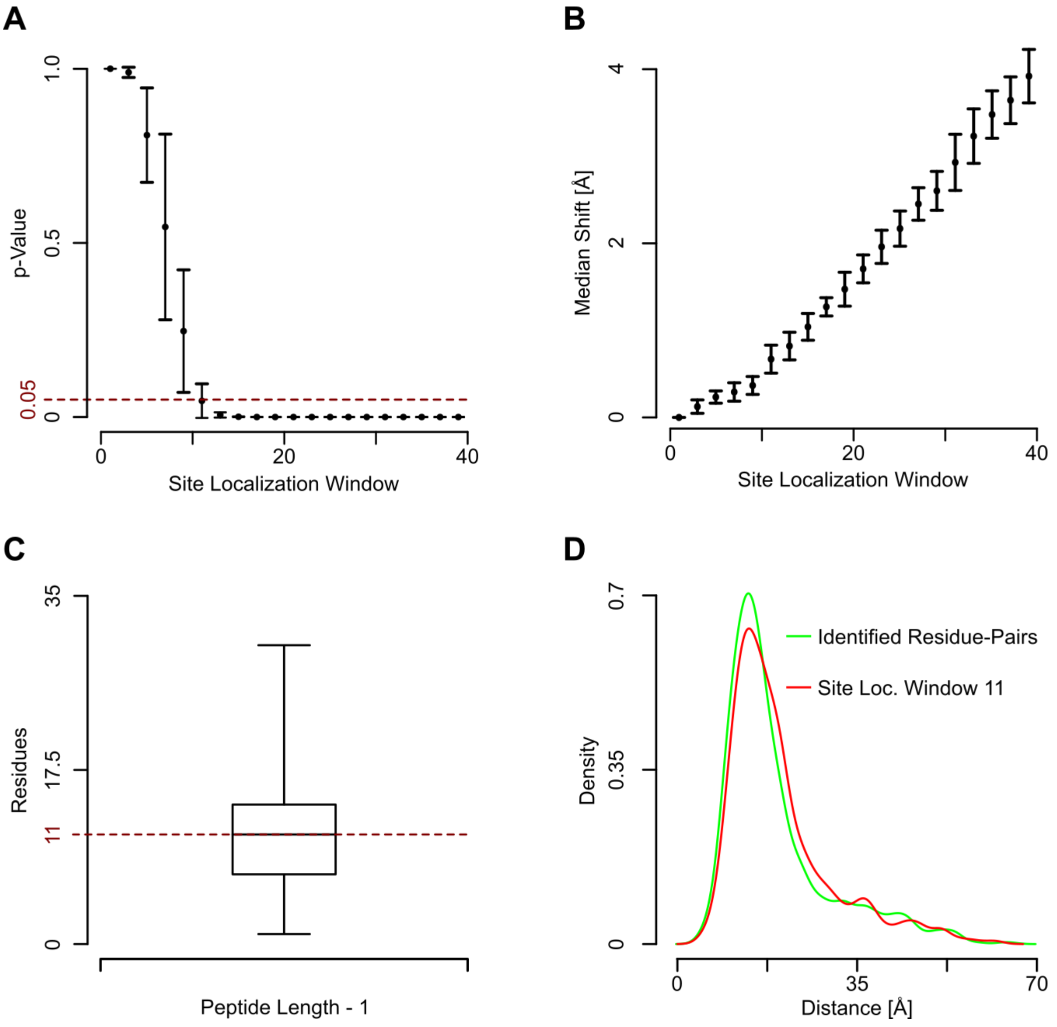

Supplemental Figure 4. Impact of Link Site Ambiguity. A: p-values for Kolmogorov-Smirnov test comparing the distance distribution of the identified residue pairs (cross-links, 20% FDR) as measured in PDB|1AO6 (alpha-carbon distances) with distance distributions

where for each unique residue pair, one site (the diazirine-linked site) was randomly reassigned to a residue within a window centered on the original match site. For each window size, 100 distributions of locally randomized residue pairs were calculated. The median p-value is plotted for each window size along with the 1.4826*MAD as error bars. **B**: The difference between the median of the original residue pair distances and the median of the randomized site distribution for each window-size. **C**: Box plot for the length - 1 of the diazirine linked peptides for all PSMs. Length -1 is used, as a link to the carboxy-terminal residue would inhibit trypsin cleavage and is therefore excluded as a possible linkage site. The median number of linkable residues is 11. **D**: Density plot of the distances of the identified residue pairs overlayed with one example of the 100 randomized distributions for a link site tolerance of 11 residues.

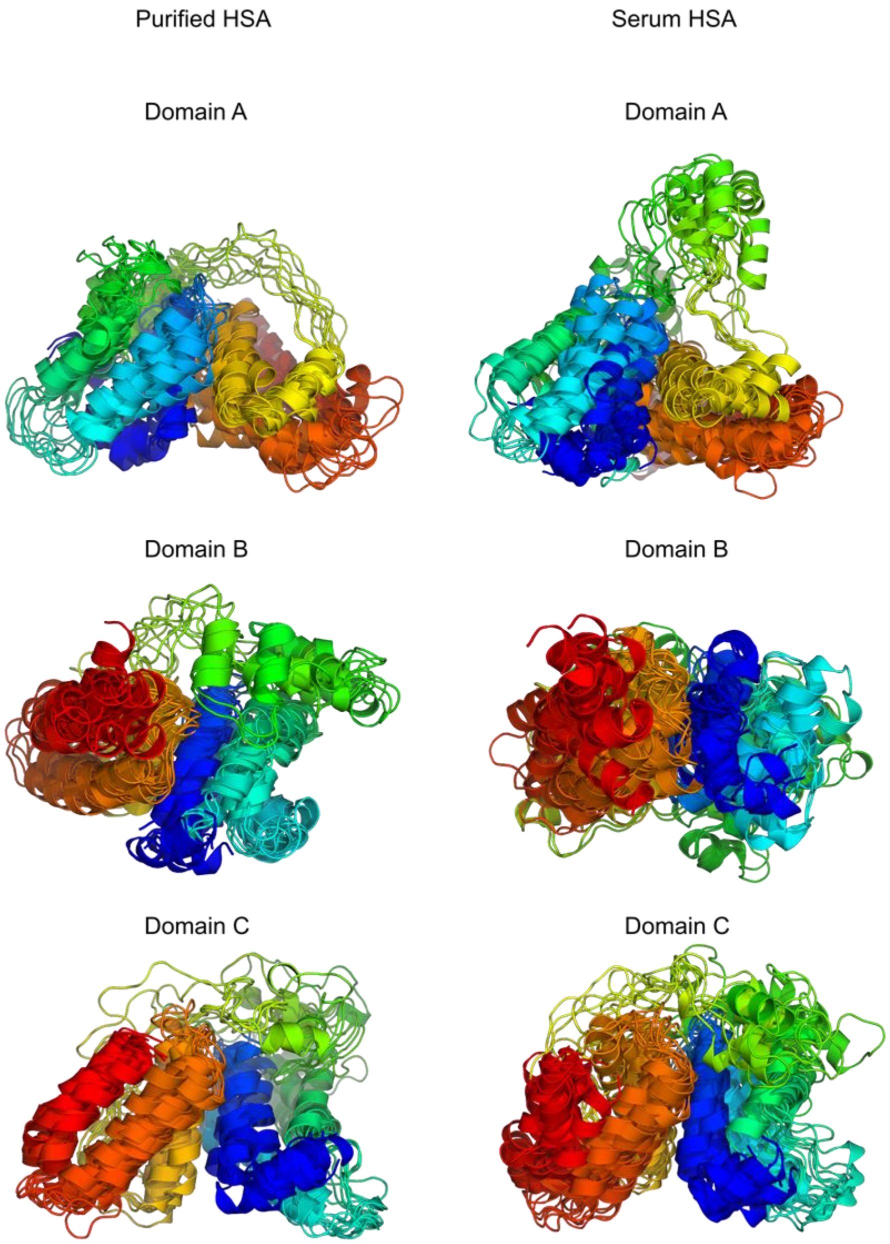

Supplemental Figure 5. Low-energy ensembles of the domains of HSA. Low-energy ensembles were computed with CLMS data from purified HSA samples (left column) and from HSA in serum (right column). For each domain, we show the 10 lowest-energy structures from our calculations. Structures in low-energy energy ensembles that are

computed from purified HSA data are in good agreement with each other, indicating convergence of the algorithm. Data from serum HSA is slightly noisier and computations produce more heterogeneous ensembles then for purified HSA. Nevertheless, the resulting ensembles show good sampling around the native state.

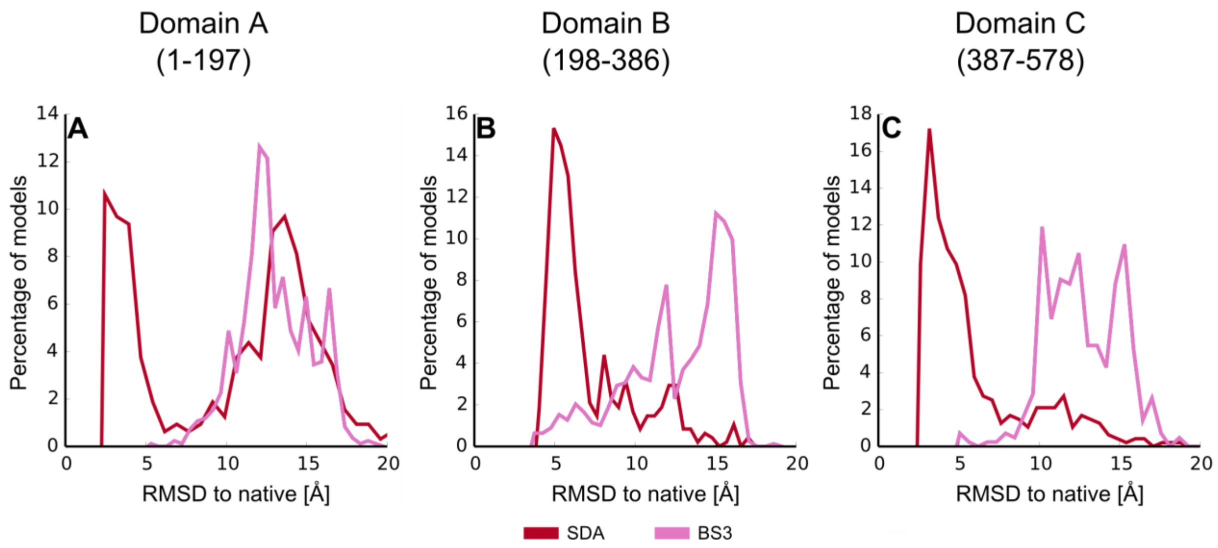

Supplemental Figure 6. Backbone quality of the structure ensemble generated with SDA cross-linker (our method) and BS3 cross-linker. We obtained both CLMS datasets from purified HSA samples. CLMS data from SDA cross-linking experiments contain an order of magnitude more cross-links (SDA: 320/107/248; BS3 16/14/11 cross-links for domains A/B/C). This leads to an increased sampling of low-RMSD HSA domain structures.

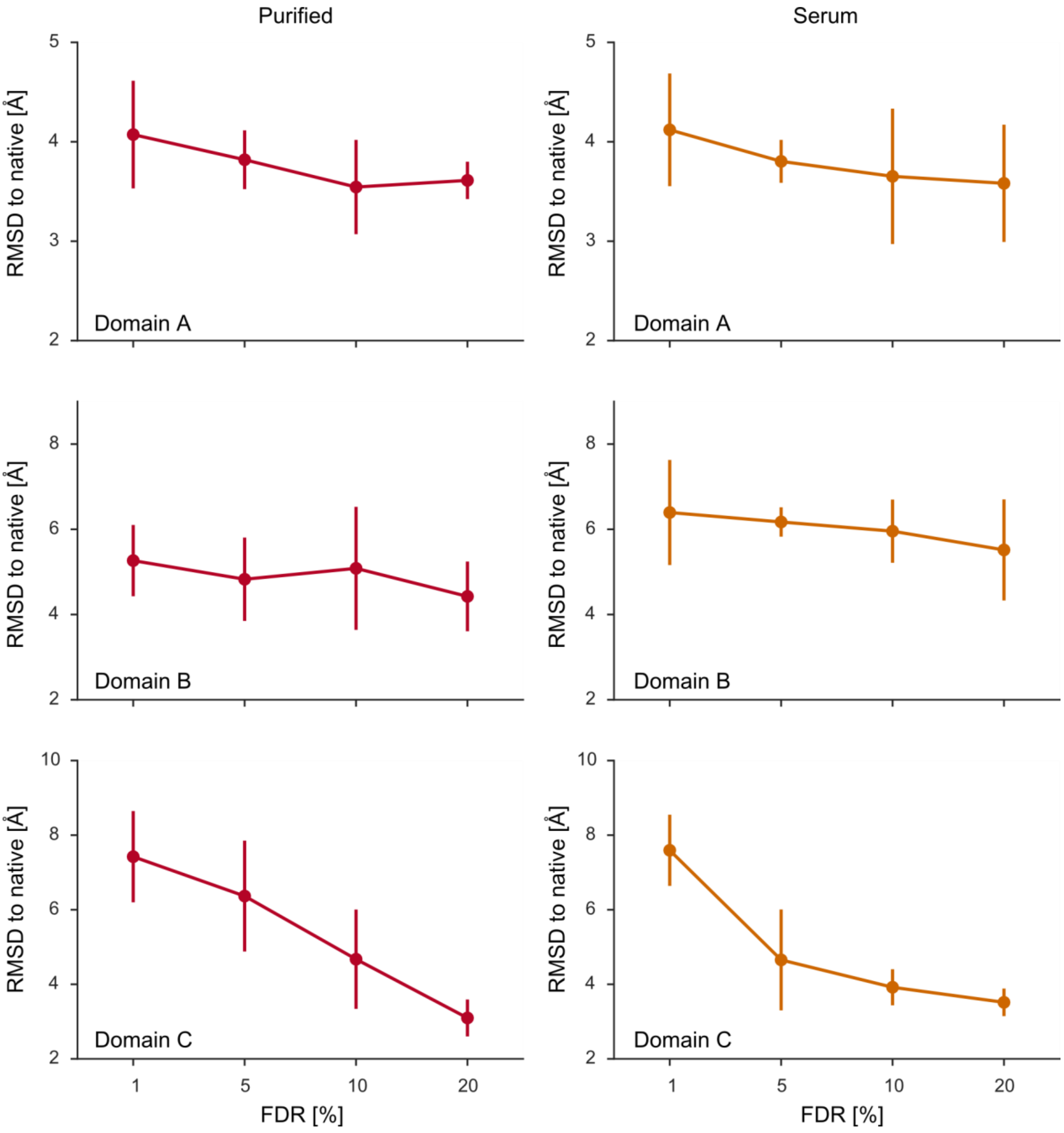

Supplemental Figure 7. Impact of the FDR on the backbone quality of the sampled structure ensemble. We repeated the modeling experiments with CLMS data at 1/5/10/20 FDR five times (see "Experimental Details"). We measured the impact on ensemble quality by the mean and standard deviation of the RMSD to native at the 1% percentile over five runs. This metric assesses the quality of the structure ensemble by the upper RMSD bound of a significant number of generated structures. Generally, CLMS data at higher FDR values improve the quality of the structure ensemble. With the exception of domain A with CLMS data from purified samples, data at 20 % FDR results in the lowest-RMSD ensemble. Note that we reduced the number of samples to 2000 samples per MBS stage (5000 for production runs in the main manuscript) for this experiment. This is necessary because of the high computational cost of this experiment.

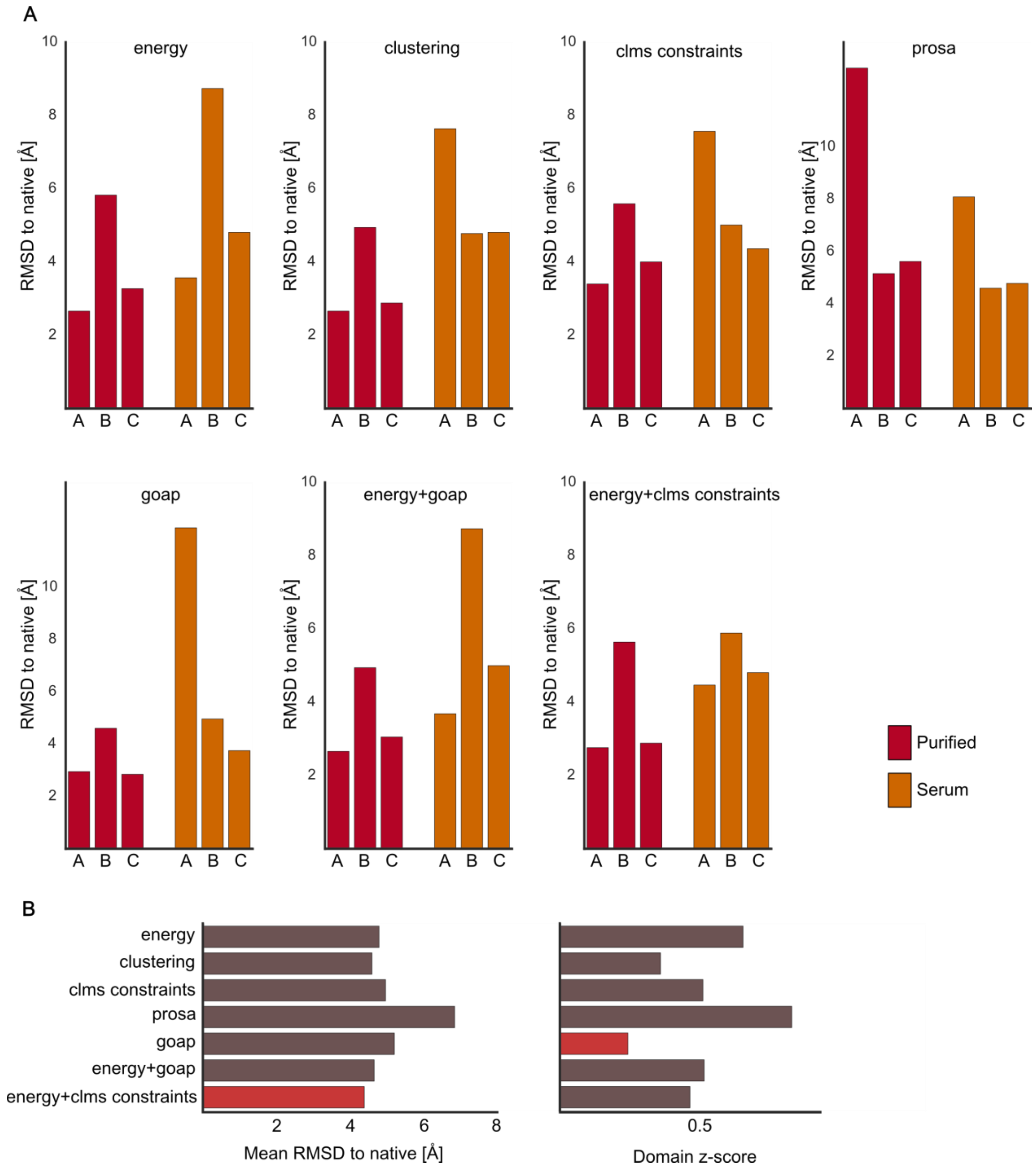

Supplemental Figure 8. RMSD of the first structure selected with several structure selection methods. **A**: Individual structure selection results for domains A/B/C with CLMS data from purified (red) and serum (orange) samples (lower is better). **B**: Overall performance (mean RMSD over all domains and domain-wise z-score) of all tested structure selection methods. The best method is shown in red. Overall, Rosetta energy+CLMS constraints selects structures with lowest mean RMSD. GOAP finds lower RMSD structures than energy+CLMS constraints for most domains, but selects a wrong fold for domain A in serum. Overall, energy+CLMS constraints does not necessarily select the best structure, but consistently selects structures with native topology (RMSD smaller than 6 Å). Therefore, energy+CLMS constraints is the most robust method we tested and is used to select the first structures in the main manuscript.

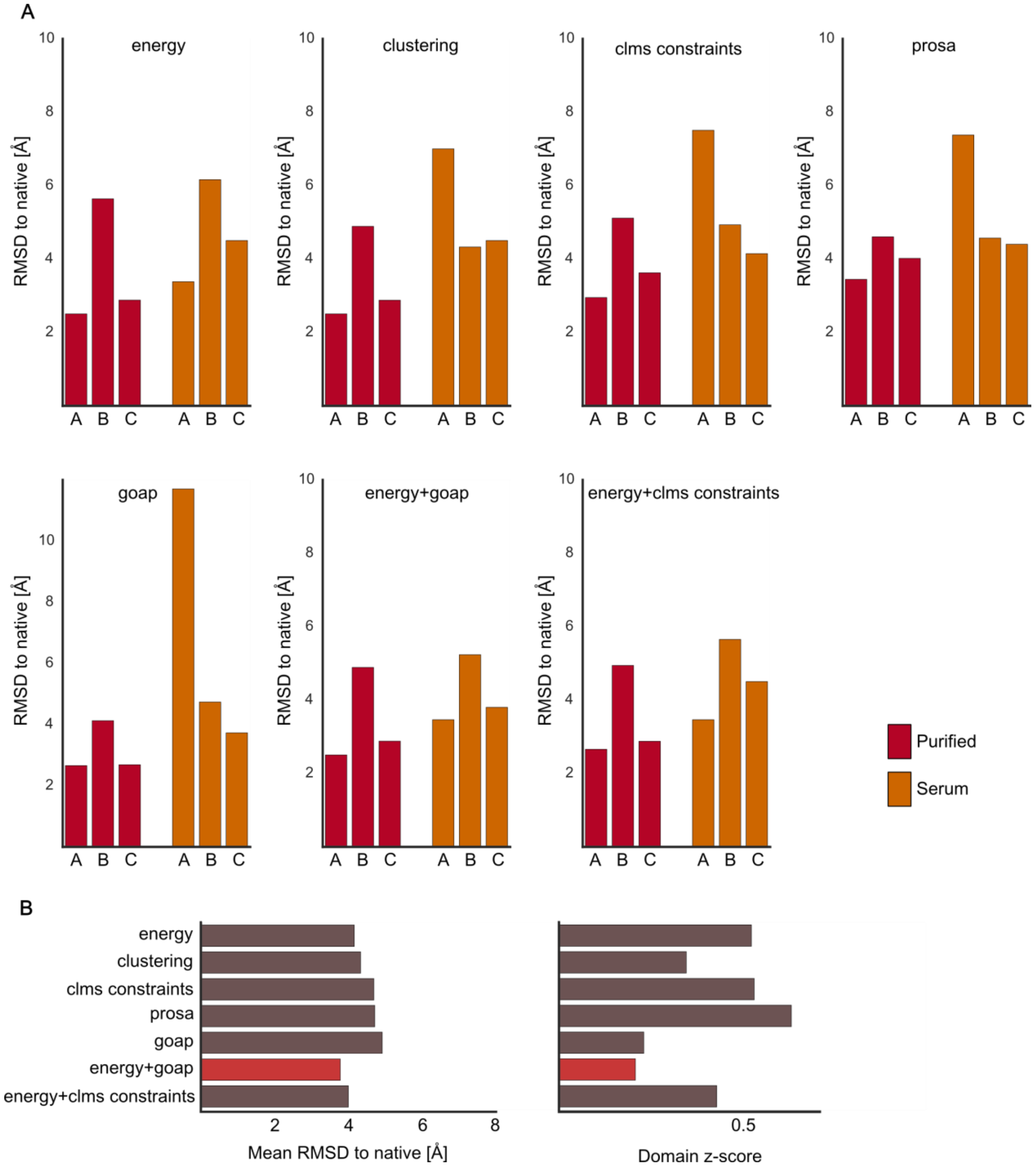

Supplemental Figure 9. RMSD of the best out of five structure selected with several structure selection methods. **A**: Individual structure selection results for domains A/B/C with CLMS data from purified (red) and serum (orange) samples (lower is better). **B**: Overall performance (mean RMSD over all domains and domain-wise z-score) of all tested decoy selection methods. The best method is shown in red. Overall, Rosetta energy+GOAP is the best method for selecting the best out of five structures. Thus, this method is able to select a small number of structures that can be further evaluated with additional experimental data and/or biological knowledge.

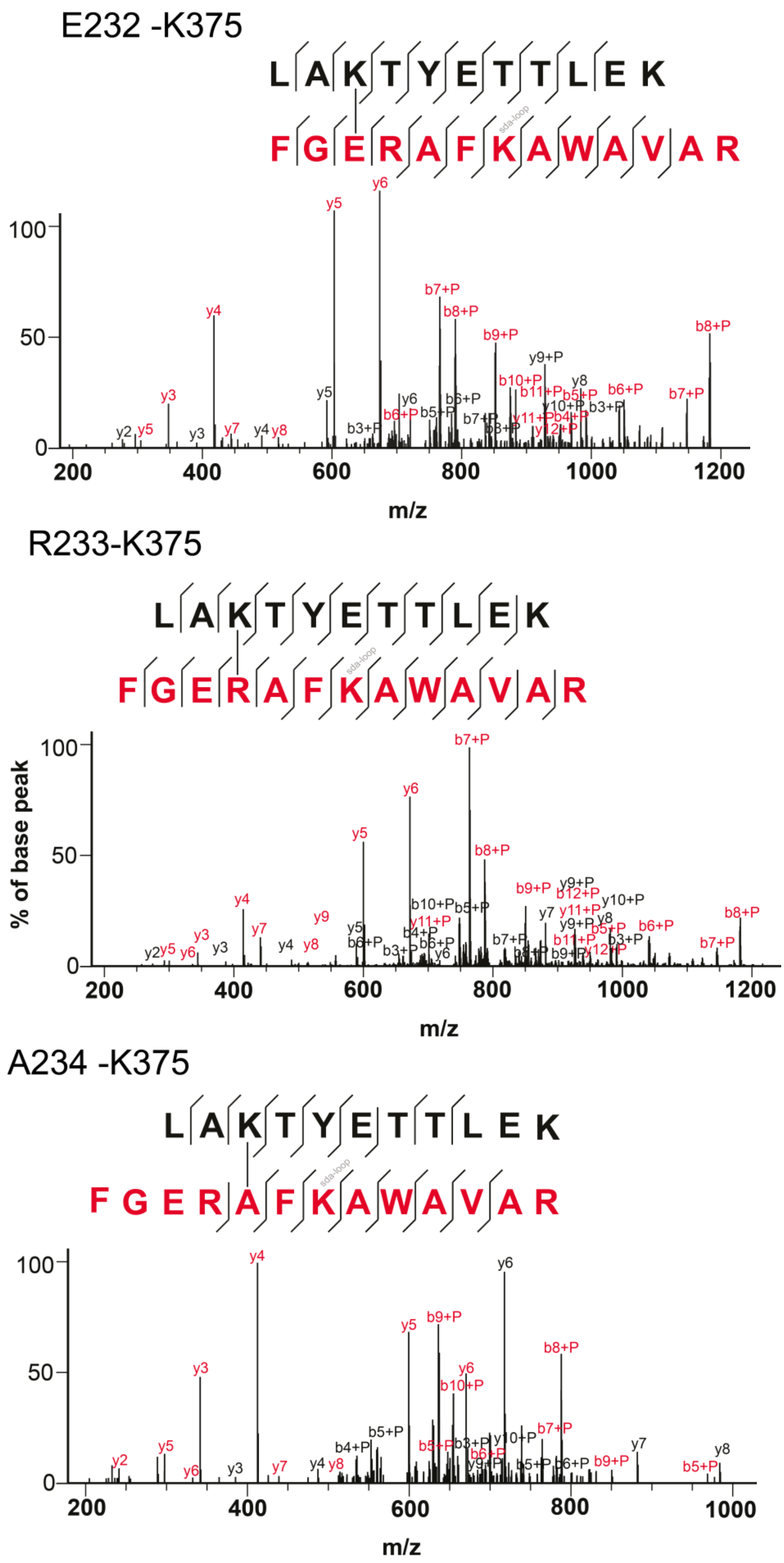

Supplemental Figure 10. Detailed spectra from Figure 3.

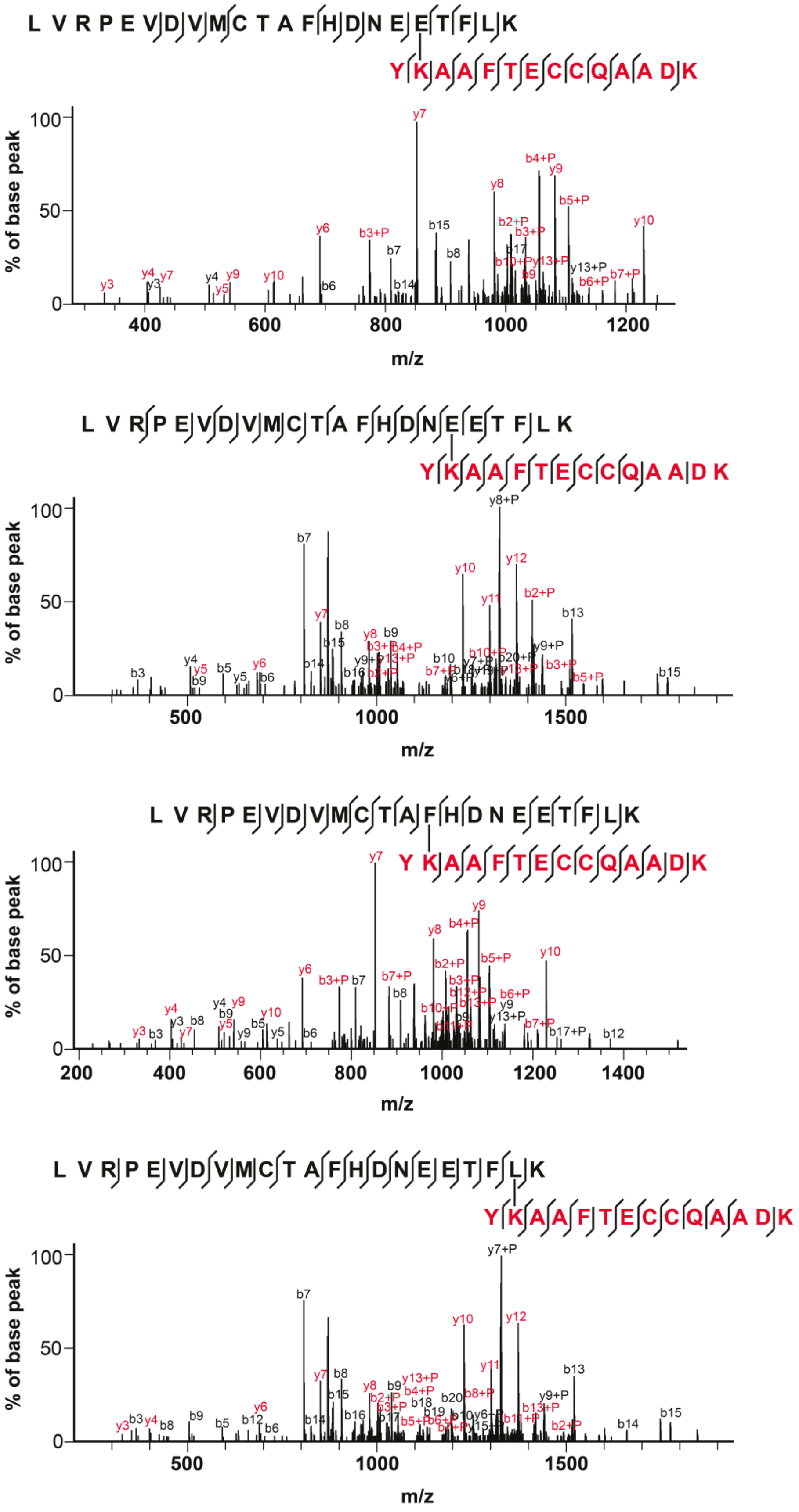
L V R P E V D V M C T A F H D N E E T F L K
Y K A A F T E C C Q A A D K
% of base peak
m/z
L V R P E V D V M C T A F H D N E E T F L K
Y K A A F T E C C Q A A D K
% of base peak
m/z
L V R P E V D V M C T A F H D N E E T F L K
Y K A A F T E C C Q A A D K
% of base peak
m/z
L V R P E V D V M C T A F H D N E E T F L K
Y K A A F T E C C Q A A D K
% of base peak
m/z

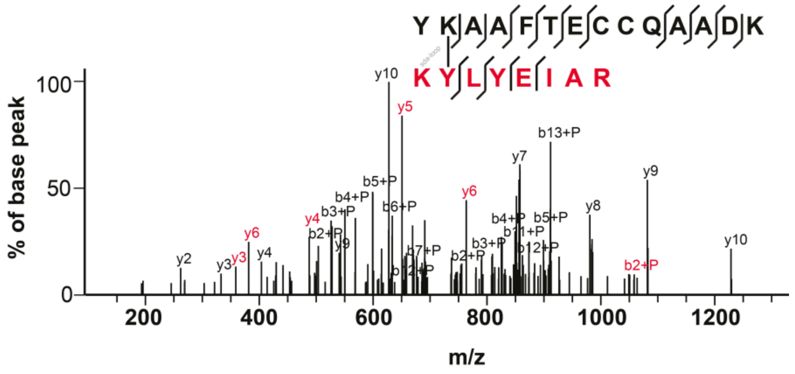

Supplemental Figure 11. Detailed spectra from Figure 4.

Supplemental Tables:

Supplemental Tables 1-4 see separate files (spreadsheets).

Supplemental Table 5: Domain boundary predictions by individual predictors.

Method	All predictions	Boundary[b] A-B	Boundary[b] B-C
DoBo	381/177/384/367/367/365/107/104[a]	177	384
ThreaDom	102/197/291/380/488[a]	197	380
PSIPRED	174/217/394[a]	217	394
Consensus		197[c]	386[c]

[a] Predictions are ranked by the corresponding method's score.
[b] Selected predictions, based on overall agreement between the methods. In case of ambiguous predictions (as for domain A), we went for the predictions with higher overall agreement in terms of residue deviation instead for the highest scoring predictions (174 with PSIPRED).
[c] The consensus is computed by averaging the selected predictions.

Supplemental Table 6: Low-energy ensembles of domains A/B/C of HSA.

Domain	Residues[a]	Residues used for full scoring/ RMSD[b]	min/median/ max[c] RMSD of purified CLMS	min/median/ max[c] RMSD of serum CLMS	min/median/ max[c] RMSD, no CLMS	min/median/ max[c] RMSD, BS3
A	1-197	2-71:115-194	2.5/3.9/7.7	3.4/4.1/10.2	7.9/12.5/ 16.6	10.2/12.1/ 16.4
B	198-386	200-262:308-381	4.9/5.7/8.4	5.2/8.3/11.5	7.4/11.8/ 14.2	11.9/15.7/ 16.5
C	387-578	389-458:508-571	2.9/3.2/5.7	3.8/5.0/8.0	15.5/16.3/ 16.9	10.4/11.1/ 17.4

[a] In our notation, the first residue in the HSA (PDB|1AO6) crystal structure is denoted as residue 1.
[b] These residues are used for full scoring in the all-atom phase and for RMSD calculation. From the non-listed residues, only repulsive terms of the energy function are used.
[c] min/median/max refers to the lowest/median/highest RMSD values in the ensemble of the ten lowest-energy structures.
